# Geographical differences of the offender-patient pathways across Europe In this part of the workshop, I will make an approach of of how will be treated an forensic psychiatric complex case from Spain point of view

**DOI:** 10.1192/j.eurpsy.2024.111

**Published:** 2024-08-27

**Authors:** V. Tort Herrando

**Affiliations:** Penitentiary Psychaitry, Parc Saniatria Sant Joan de Deu, Sant Boy de Llobregat (Barcelona), Spain

## Abstract

**Abstract:**

Geographical differences of the offender-patient pathways across Europe

In this part of the workshop,I will make an approach of of how will be treated an forensic psychiatric complex case from Spain point of view, and diffenneces with another countries.  . Differents pathways from detention to be admitedd in a psychiatric facility will be described . Also the approach from standard care to a more complex medical situacion ( from clinical, social and psychological viewa ) in a penitentiary (forensic) resources, including  rehablitaation. And finally, the follow-up / after care of a mentally ill offender, when discharge to the commnuity.

**Disclosure of Interest:**

None Declared

